# Adherence to the Mediterranean diet as a possible additional tool to be used for screening the metabolically unhealthy obesity (MUO) phenotype

**DOI:** 10.1186/s12967-023-04546-0

**Published:** 2023-09-28

**Authors:** Luigi Barrea, Ludovica Verde, Daniel Simancas-Racines, Ana Karina Zambrano, Evelyn Frias-Toral, Annamaria Colao, Silvia Savastano, Giovanna Muscogiuri

**Affiliations:** 1 Dipartimento di Scienze Umanistiche, Centro Direzionale, Università Telematica Pegaso, Via Porzio, Isola F2, 80143 Naples, Italy; 2 Centro Italiano Per la cura e il Benessere del Paziente con Obesità (C.I.B.O), Diabetologia e Andrologia, Dipartimento di Medicina Clinica e Chirurgia, Unità di Endocrinologia, Università degli Studi di Napoli Federico II, Via Sergio Pansini 5, 80131 Naples, Italy; 3 Department of Public Health, University of Naples Federico II, Via Sergio Pansini 5, 80131 Naples, Italy; 4 Centro de Investigación en Salud Pública y Epidemiología Clínica (CISPEC), Facultad de Ciencias de la Salud Eugenio Espejo, Universidad Tecnológica Equinoccial, Quito, Ecuador; 5 School of Medicine, Universidad Católica Santiago de Guayaquil, Guayaquil, Guayas Ecuador; 6 Dipartimento di Medicina Clinica e Chirurgia, Diabetologia ed Andrologia, Unità di Endocrinologia, Università Federico II, Via Sergio Pansini 5, 80131 Naples, Italy; 7 Cattedra Unesco “Educazione Alla Salute E Allo Sviluppo Sostenibile”, University Federico II, Naples, Italy

**Keywords:** Mediterranean diet, PREDIMED, Metabolically healthy obesity, MHO, Metabolically unhealthy obesity, MUO, Obesity, Diet, Nutrition

## Abstract

**Background:**

The terms metabolically healthy obesity (MHO) and metabolically unhealthy obesity (MUO) categorize subjects with obesity based on the presence or absence of cardio-metabolic risk factors. Detecting MUO phenotype is crucial due to the high risk of cardio-metabolic complications, requiring tailored and intensive follow-up. However, diagnosing MUO is time-consuming and costly. Thus, we aimed to investigate the role of Mediterranean diet (MD) in determining MHO/MUO phenotypes and whether adherence to MD could serve as an additional screening tool for MUO phenotype.

**Methods:**

The study population of this cross-sectional observational study consisted of 275 subjects with obesity. We assessed their lifestyle habits (physical activity and smoking habits), anthropometric measurements (weight, height, waist circumference, body mass index), blood pressure, metabolic parameters, inflammatory marker (high sensitivity C reactive protein levels), adherence to MD (by *PREvención con DIetaMEDiterránea* (PREDIMED) questionnaire), and MHO/MUO phenotypes.

**Results:**

The study included 275 individuals with obesity (256F/19M; 34.0 ± 10.5 years; BMI 38.3 ± 5.95 kg/m^2^). Among them, 114 (41.5%) exhibited MHO phenotype, while 161 (58.5%) had MUO phenotype. MHO phenotype exhibited favorable anthropometric and cardio-metabolic profiles, characterized by lower waist circumference (p < 0.001), BMI (p < 0.001), insulin resistance (p < 0.001), blood pressure (p < 0.001), inflammation (p < 0.001), and lipid levels (p < 0.001) compared to MUO phenotype. Notably, we found that MHO phenotype had higher adherence to MD (p < 0.001) and consumed more extra virgin olive oil (EVOO) (p < 0.001), vegetables (p < 0.001), fruits (p < 0.001), legumes (p = 0.001), fish (p < 0.001), wine (p = 0.008), and nuts (p = 0.001), while reporting lower intake of red/processed meats (p < 0.001), butter, cream, margarine (p = 0.008), soda drinks (p = 0.006), and commercial sweets (p = 0.002) compared to MUO phenotype. Adherence to MD (p < 0.001) and EVOO (p = 0.015) intake were identified as influential factors in determining the presence of MUO/MHO phenotypes. Furthermore, a PREDIMED score < 5 proved to be the most sensitive and specific cut-point value for predicting the presence of MUO phenotype (p < 0.001).

**Conclusion:**

High adherence to MD was associated with MHO phenotype. Moreover, we suggest that a specific cut-off of the PREDIMED score could be an indicator to discriminate patients with MUO/MHO phenotypes and therefore help in identifying patients at higher cardiovascular risk who will require specific dietary intervention.

## Introduction

Obesity is a pressing issue of worldwide concern, with a substantial impact on public health and healthcare costs [1]. It is estimated that around 13% of the global population is affected by this chronic, progressive, and relapsing disease [1]. Of note, suffering from obesity is linked to a heightened likelihood of experiencing diverse metabolic issues and long-term illnesses [2]. These include conditions like type 2 diabetes, cardiovascular disorders, and specific cancers such as breast and colon cancer, as well as a reduced overall lifespan [2]. Moreover, recent evidence suggests that obesity is a risk factor for sleep disorders and reduced sleep quality [3–5]. However, despite all these risks, there are subjects with obesity who do not exhibit metabolic abnormalities. This is because relying solely on body mass index (BMI) doesn't encompass the heightened risk linked to varying patterns of body fat distribution [6]. Specifically, the inflammatory state resulting from the accumulation of visceral adipose tissue plays a role in promoting insulin resistance in adipose tissue, skeletal muscle, and the liver, as well as contributing to features of metabolic syndrome (MetS) [7]. As a result, the terms metabolically healthy obesity (MHO) and metabolically unhealthy obesity (MUO) have been introduced to categorize subjects with obesity complicated or not by cardio-metabolic risk factors, respectively [8].

Numerous studies have demonstrated MHO phenotype has a lower risk of developing type 2 diabetes, cardiovascular disease, and premature mortality compared to MUO phenotype [9–11].

Due to the absence of a universally accepted definition for MHO, which in turn leads to differences in the prevalence of this specific metabolic profile associated with obesity, a meta-analysis of 19 studies reported that approximately 35% of subjects with obesity were metabolically healthy, with variations across different countries [12]. Interestingly, MUO phenotype could be considered an “oxidative stress-related condition” because both total and mitochondrial reactive oxygen species (ROS) production are enhanced compared to MHO phenotype [13].

In addition, the metabolic phenotype of obesity is influenced by adipose tissue distribution, which plays a central role in promoting metabolic and cardiovascular deterioration [14]. MUO phenotype demonstrates adipocyte hypertrophy, elevated accumulation of visceral and subcutaneous abdominal adipose tissue, and disproportionate lipid deposition in organs like the liver and skeletal muscle [15]. These characteristics are accompanied by increased inflammation, macrophage infiltration, and altered secretion of adipokines from the hypertrophic adipose tissue [15]. Collectively, these disturbances contribute to the development of peripheral insulin resistance [16], which is closely associated with metabolic and cardiovascular alterations [7].

Given this evidence, it is of paramount importance to identify MUO phenotype because these subjects are at high risk of developing cardiometabolic complications and thus need a tailored and tighter follow-up. However, the diagnosis of MUO phenotype takes a long time and can be expensive for the National Health System since it requires the assessment of multiple biochemical parameters.

Considerable evidence suggests that adopting a healthy lifestyle can lower the risk of cardio-metabolic issues, regardless of its impact on body weight [17]. Mediterranean diet (MD) is characterized by a substantial consumption of vegetables, fruits, whole grains, legumes, and nuts. It also entails a moderate approach to dairy consumption, restricted intake of meat and poultry, and a moderate inclusion of alcohol, such as red wine served during meals [18]. Acknowledging its cultural importance, UNESCO added this diet to the Representative List of Intangible Cultural Heritage in 2010 [19]. Thus, we aim to investigate: [1] if MD has a role in determining MUO phenotype; [2] if adherence to MD could be an additional tool to be used to screen MUO phenotype.

## Methods

### Design, setting and population study

We performed a cross-sectional observational study involving participants enrolled at the Department of Clinical Medicine and Surgery; Endocrinology, Diabetology, and Andrology Unit, University of Naples Federico II. This study was conducted from January 2016 to January 2023. The gathered data were recorded in an electronic medical system and, following the acquisition of signed informed consent, transferred into a comprehensive database. This anonymized dataset was subsequently utilized for research endeavors.

**Fig. 1:**
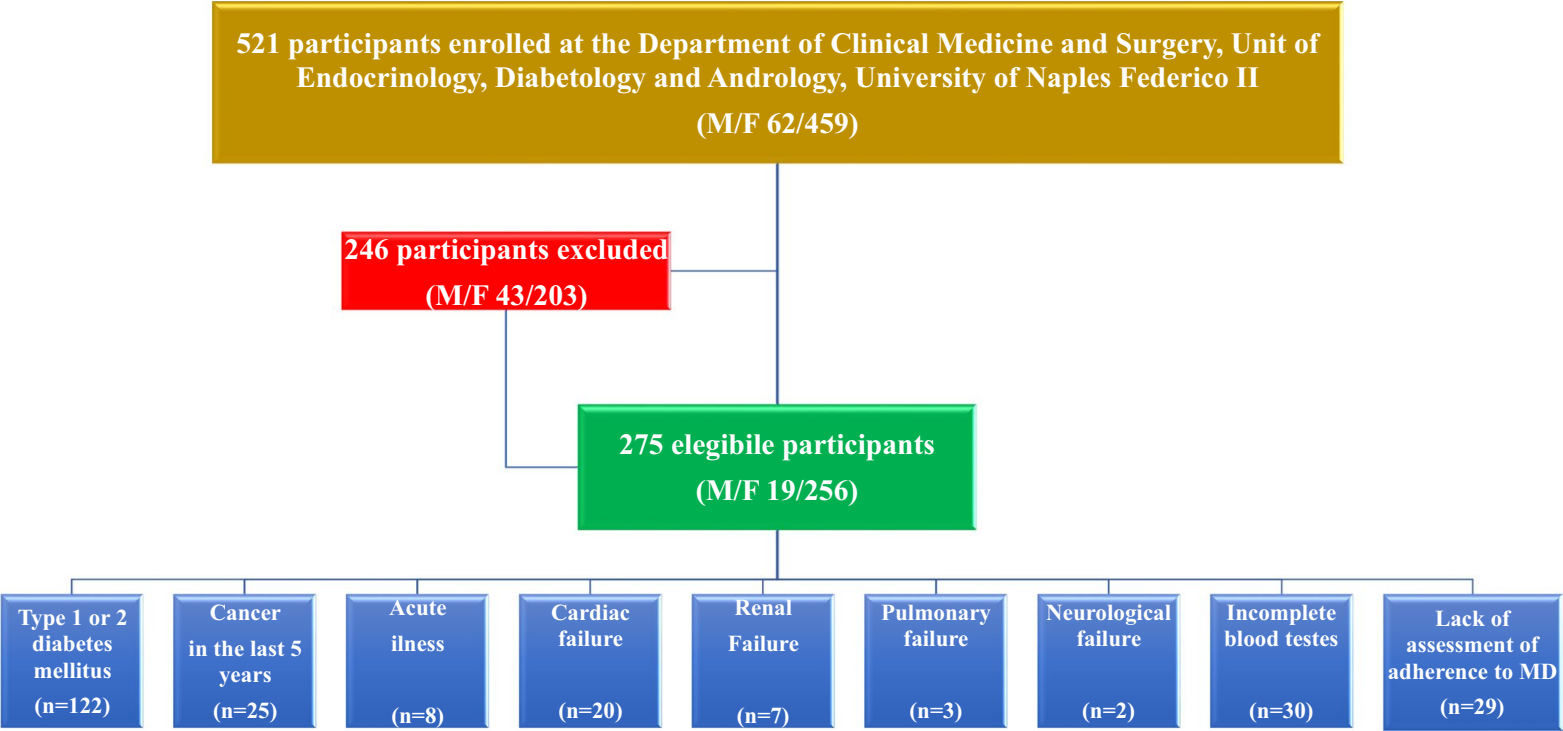
Flow chart of the study participants

For this research, we included subjects with obesity (BMI ≥ 30.0 kg/m^2^) of both sexes, aged 18 years or older, who hadn't been previously diagnosed with type 1 or 2 diabetes, cancer in the last 5 years, gastrointestinal, neurological, renal, cardiac, and pulmonary failure, or acute illness. To be eligible for the study, all subjects were required to undergo a medical examination, anthropometric evaluation, blood sampling, and evaluation of adherence to MD. The research adhered to the principles outlined in the Declaration of Helsinki, and the Ethics Committee at Federico II University of Naples provided a favorable assessment of the study protocols (protocol no. 05/14). All participants had to provide written, informed consent. After obtaining written informed consent, 521 participants were consecutively enrolled. According to the exclusion criteria, a total of 275 participants remained for analysis. The flow diagram of the studied participants is shown in Fig. 1.

### Anthropometric measurements

Anthropometric measurements were taken in the early morning hours, between 8 a.m. and 10 a.m., after an overnight fasting period. An experienced nutritionist performed these measurements using established protocols. Specifically, during the examination, participants were instructed to wear light clothing and not to wear shoes. The subjects' height and weight were measured to calculate their BMI, which consists of dividing weight in kilograms by height squared in square meters (kg/m^2^). Height (meters) was assessed with a wall stadiometer (Seca 711; Seca, Hamburg, Germany) with an accuracy of 0.5 cm, while body weight (kilograms) was determined with a calibrated scale (Seca 711; Seca, Hamburg, Germany) with an accuracy of 0.1 kg. The waist circumference (WC) in centimeters was measured to the nearest 0.1 cm using an inelastic measuring tape, either at the natural indentation or halfway between the lower edge of the rib cage and the iliac crest in cases where a natural indentation could not be detected.

### Adherence to Mediterranean diet

Adherence to MD was evaluated using the PREDIMED questionnaire, which had undergone validation previously [20]. As documented in earlier studies [21–24], a nutritionist with specialized training conducted face-to-face interviews with all participants to administer the questionnaire. Each question was allocated a score of either 1 or 0, and the overall PREDIMED score was determined by summing these scores [20]. A score falling within the range of 0 to 5 indicated the least adherence to MD; a score of 6 to 9 denoted moderate adherence; and a score of 10 or higher signified the highest degree of adherence to MD [20].

### Physical activity and smoking habits

Physical activity levels were gauged through a standardized questionnaire, querying participants about their regular engagement in at least 30 min of aerobic exercise daily (with yes or no responses). In a similar vein, smoking behaviors were also assessed using a standardized questionnaire (with yes or no responses). Individuals who had ceased smoking at least one year prior to the interview were categorized as ‘‘former smokers’’, while those who smoked a minimum of one cigarette daily were classified as ‘‘current smokers’’. Participants who were neither current smokers nor had smoked in the previous year were designated as ‘‘non-current smokers’’. For analytical purposes, individuals categorized as either former or non-current smokers were combined into a group termed ‘‘non-smokers.’’ The procedures outlined have been employed in preceding investigations [21–23].

### Blood pressure assessment

Diastolic blood pressure (DBP) and systolic blood pressure (SBP) were assessed in all participants. As documented in an earlier publication [25], this was done by conducting three separate measurements using a random zero sphygmomanometer (Gelman Hawksley Ltd., Sussex, UK). The measurements were taken after the subjects had been seated and resting for a duration of 10 min. The final reported value was the average of the last two measurements taken.

### Assay methods

Samples were gathered during the morning hours, specifically between 8 and 10 a.m., after the subjects had fasted for a minimum of 8 h. These specimens were then preserved at a temperature of − 80 °C until processing. Fasting plasma glucose (mg/dL), total cholesterol (mg/dL), and triglycerides (mg/dL) were evaluated using the Roche Modular Analytics System in the Central Biochemistry Laboratory of the institution. For quantifying levels of high-density lipoprotein (HDL) and low-density lipoprotein (LDL) cholesterol (mg/dL), a direct method utilizing a homogeneous enzymatic assay was employed. Additionally, fasting plasma insulin (μU/mL) levels were determined using commercially available kits and a solid-phase chemiluminescent enzyme immunoassay. The reported intra-assay coefficients of variation (CVs) were below 5.5%, consistent with earlier findings [21–23].

### Insulin resistance assessment

The Homeostatic Model Assessment of Insulin Resistance (HoMA-IR) was computed following the method described by Matthews et al. [26].

$${\text{HoMA}}\,{\text{ - }}\,{\text{IR = }}[({\text{fasting}}{\mkern 1mu} {\text{plasma glucose}}{\mkern 1mu} {\text{(mg/dL)}} \times {\text{fasting plasma insulin (}}\mu {\text{U/mL)/405}}$$

This assessment is a means of estimating insulin resistance. Specifically, a HoMA-IR value exceeding 2.5 was employed as a threshold to identify the presence of insulin resistance [26].

### High-sensitivity C-reactive protein level assessment

Blood samples were obtained from veins in the morning hours, specifically between 8 and 10 a.m., following a fasting period of at least 8 h. Levels of high-sensitivity C-reactive protein (hs-CRP) were determined using a high-sensitivity nephelometric assay (CardioPhase hsCRP kit, Siemens Healthcare Diagnostics, Marburg, Germany). The assay had a minimum detection limit of 0.01 mg/L, and both the within-assay and between-assay CVs were less than 7%. In accordance with guidelines from the CDC and the AHA, the participants were classified into three categories based on their hs-CRP levels: high cardiovascular risk (≥ 3.0 mg/L), intermediate cardiovascular risk (1.0–3.0 mg/L), and low cardiovascular risk (< 1.0 mg/L) [27]. This classification helps determine the subjects' potential risk of cardiovascular issues based on their hs-CRP levels.

### Metabolic healthy obesity and metabolic unhealthy obesity assessment

MetS was diagnosed following the criteria outlined by the NCEP ATP III definition [28] (as detailed in Table 1). To be more specific, as per the ATP-III definition, individuals were considered metabolically normal if they had either one or none of the subsequent components: triglycerides ≥ 150 mg/dL or were using lipid-lowering medications; SBP ≥ 130 mmHg or DBP ≥ 85 mmHg or were using antihypertensive medications; fasting plasma glucose ≥ 110 mg/dL or were using medications for type 2 diabetes mellitus; and HDL cholesterol < 50 mg/dL for women and < 40 mg/dL for men. It’s worth noting that the WC criterion wasn’t used due to its potential correlation with BMI. As previously reported, subjects that fulfilled less than two of the MetS criteria were considered MHO phenotypes, conversely, the remainder were classified as MUO phenotypes [29].

**Table 1:** National cholesterol education program adult treatment panel (NCEP ATP) III criteria for MetS

Parameters	Criteria for MetS
Impaired glucose tolerance	Fasting plasma glucose ≥ 100 mg/dL (5.6 mmol/L)
Abdominal obesity	WC > 102 cm in men WC > 88 cm in women
Hypertriglyceridemia	≥ 150 mg/dL (1.7 mmol/L) or drug treatment for high triglycerides
Low levels of HDL cholesterol	< 40 mg/dL (1 mmol/L) in men < 50 mg/dL (1.3 mmol/L) in women or drug treatment for low HDL cholesterol
High blood pressure	≥ 130/85 mmHg or drug treatment for hypertension

*MetS* metabolic syndrome, *WC* waist circumference, *HDL* high-density lipoprotein

### Statistical analysis

Categorical variables were reported as numbers (n) and percentages (%) whereas continuous variables were expressed as the mean ± SD. The Kolmogorov–Smirnov test was used to test data distribution. Skewed parameters (triglycerides, total, and LDL cholesterol) were normalized by logarithm transformation and reconverted into tables and figures. Differences in sex, age, lifestyle habits, anthropometric measurements, blood pressure, metabolic parameters, inflammatory parameters, and nutritional parameters between MHO and MUO phenotypes were analyzed by the Student’s independent *t*-test. The chi square (χ^2^) test was used to determine the significance of differences in frequency distributions of categorical variables. Proportional odds ratio (OR) models were performed to assess the association of MHO and MUO phenotypes with dietary components of the PREDIMED questionnaire. Correlations between study variables were performed using Pearson’s *r* correlation coefficients (continuous variables). A logistic regression, expressed as R^2^, beta (*β*), and *t*, with MHO and MUO phenotypes as dependent variables, was used to estimate the predictive value of the dietary components included in the PREDIMED questionnaire and the PREDIMED score. Receiver operator characteristic (ROC) curve analysis was performed to determine sensitivity and specificity, area under the curve (AUC), as well as cut-off values of the PREDIMED score in detecting MUO phenotype. A *p* value < 0.05 was considered significant. Statistical analysis was performed according to standard methods using the Statistical Package for Social Sciences software 26.0 (SPSS/PC; SPSS, Chicago, IL, USA).

## Results

The study population consisted of 275 subjects with obesity (256 F/19 M; 34.0 ± 10.5 years; BMI: 38.3 ± 5.95 kg/m^2^). Table 2 reports sex, age, lifestyle habits, anthropometric measurements, blood pressure, metabolic and inflammatory parameters, and the number of MetS parameters analyzed in this study. Most of the subjects were female (93.1%), sedentary (68.7%), and not smokers (60.0%). Adherence to MD was low on average (PREDIMED score 5.75 ± 2.27). Most of the participants had grade II obesity (36.0%). The mean WC was 115.05 ± 17.68 cm while mean SBP and DBP were (130.20 ± 12.38 mmHg) and (81.80 ± 9.21 mmHg), respectively. More than 50% of the participants were insulin-resistant (HoMA-IR > 2.5). More than 30% of patients had hs-CRP levels higher than 3.0 mg/L, representative of a high risk of cardiovascular disease.

**Table 2:** Parameters analyzed in the study population

Parameters (N = 275)	Mean ± SD or n (%)
Sex	
Male (n, %)	19 (6.9)
Female (n, %)	256 (93.1)
Age (years)	34.01 ± 10.60
Lifestyle habits	
Physical activity	
Yes (n, %)	86 (31.3)
No (n, %)	189 (68.7)
Smoking	
Yes (n, %)	110 (40.0)
No (n, %)	165 (60.0)
PREDIMED score	5.75 ± 2.27
Anthropometric measurements	
BMI (kg/m^2^)	38.39 ± 5.96
Obesity I grade (n, %)	90 (31.7)
Obesity II grade (n, %)	99 (36.0)
Obesity III grade (n, %)	86 (31.3)
WC (cm)	115.05 ± 17.68
Blood pressure	
SBP (mmHg)	130.20 ± 12.38
DBP (mmHg)	81.80 ± 9.21
Metabolic parameters	
Fasting plasma glucose (mg/dL)	101.32 ± 15.60
Fasting plasma insulin (μU/mL)	16.15 ± 14.63
HoMA-IR	4.24 ± 4.10
< 2.5 (n, %)	123 (44.7)
> 2.5 (n, %)	152 (55.3)
Total cholesterol (mg/dL)	206.19 ± 39.93
LDL cholesterol (mg/dL)	133.04 ± 39.81
HDL cholesterol (mg/dL)	42.30 ± 11.59
Triglycerides (mg/dL)	154.27 ± 49.14
Inflammatory parameters	
hs-CRP (mg/L)	2.90 ± 2.50
Low cardiovascular risk (< 1.0 mg/L) (n, %)	60 (21.8)
Intermediate cardiovascular risk (1.0 – 3.0 mg/L) (n, %)	123 (44.7)
High cardiovascular risk (> 3.0 mg/L) (n, %)	92 (33.5)
Metabolic syndrome	
Number parameters	3.01 ± 1.41

Data are expressed as number and percentage or mean ± SD

*BMI*, body mass index, *WC* Waist circumference, *SBP* systolic blood pressure, *DBP* diastolic blood pressure, *HoMA-IR* homeostasis model assessment insulin resistance *LDL* low-density lipoprotein, *HDL* high-density lipoprotein, *SD* standard deviation, *hs-CRP* high sensitivity C reactive protein

**Fig. 2:**
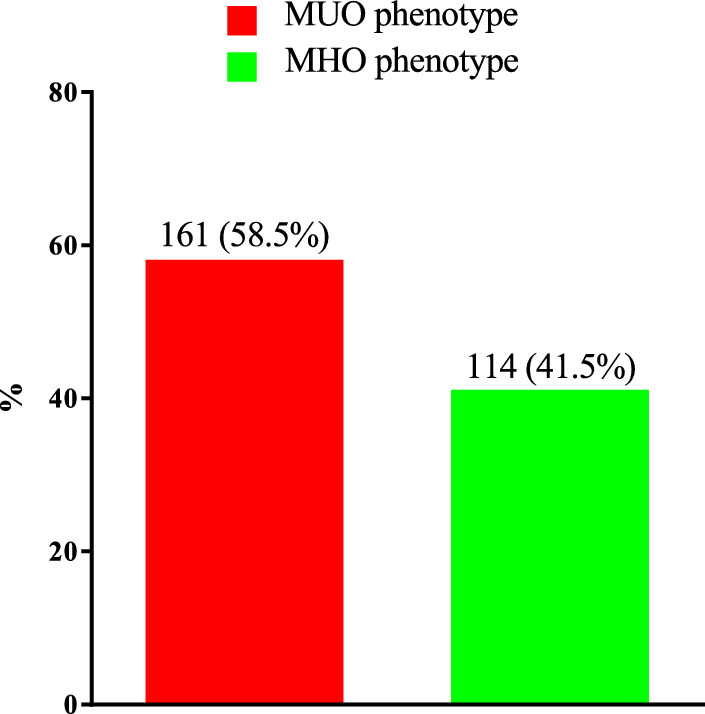
MUO/MHO phenotype in the study population. *MHO* metabolic healthy obesity, *MUO* metabolic unhealthy obesity.

One hundred and fourteen (41.5%) subjects had the MHO phenotype, and 161 (58.5%) had the MUO phenotype; see Fig. 2.

Table 3 reports the response frequency of dietary components included in PREDIMED questionnaire and adherence to MD of the entire study population. One hundred and thirty subjects (47.2%) had low adherence to MD, 136 (49.5%) had intermediate adherence to MD and 9 (3.3%) had high adherence to MD.

**Table 3:** Response frequency of dietary components included in PREDIMED questionnaire and adherence to MD of the entire study population

Questions PREDIMED questionnaire (N = 275)	n	%
Use of EVOO as main culinary lipid	187	68.0
EVOO > 4 tablespoons	126	45.8
Vegetables ≥ 2 servings/day	135	49.1
Fruits ≥ 3 servings/day	101	36.7
Red/processed meats < 1/day	93	33.8
Butter, cream, margarine < 1/day	120	43.6
Soda drinks < 1/day	126	45.8
Wine glasses ≥ 7/week	120	43.6
Legumes ≥ 3/week	102	37.1
Fish/seafood ≥ 3/week	99	36.0
Commercial sweets and confectionery ≤ 2/week	84	30.5
Tree nuts ≥ 3/week	95	34.5
Poultry more than red meats	76	27.6
Use of sofrito sauce ≥ 2/week	119	43.3
PREDIMED categories		
Low adherence to MD	130	47.2
Average adherence to MD	136	49.5
High adherence to MD	9	3.3

*PREDIMED* PREvención con DIetaMEDiterránea, *MD* Mediterranean Diet, *EVOO* extra virgin olive oil

Table 4 reports the differences in anthropometric measurements, blood pressure, metabolic, inflammatory, and nutritional parameters according to MHO or MUO phenotype. Comparing MHO phenotype to MUO phenotype, the latter had significantly higher BMI (p < 0.001), WC (p < 0.001), hs-CRP (p < 0.001), fasting plasma glucose (p < 0.001), fasting plasma insulin (p < 0.001), HoMA-IR (p < 0.001), total cholesterol (p < 0.001), LDL cholesterol (p < 0.001), and triglycerides (p < 0.001) values. MHO phenotype had significantly higher HDL cholesterol values (p < 0.001) and PREDIMED score (p < 0.001) than MUO phenotype.

**Table 4:** Parameters analyzed in the study population according to MHO or MUO phenotype

Parameters	MHO phenotype n = 114, 41.4%	MUO phenotype n = 161, 58.5%	*p-value
Sex			
Male (n, %)	10 (8.8%)	9 (5.6%)	χ^2^ = 0.61, 0.433
Female (n, %)	104 (91.2%)	152 (94.4%)	
Age (years)	33.55 ± 12.24	32.92 ± 9.15	0.053
Lifestyle habits			
Physical activity			
Yes (n, %)	39 (34.2%)	47 (29.2%)	χ^2^ = 0.57, 0.452
No (n, %)	75 (65.8%)	114 (70.8%)	
Smoking			
Yes (n, %)	49 (43.0%)	61 (37.9%)	χ^2^ = 0.53, 0.469
No (n, %)	65 (57.0%)	100 (62.1%)	
Anthropometric measurements			
BMI (kg/m^2^)	36.53 ± 4.58	39.70 ± 6.47	** < 0.001**
Obesity I grade (n, %)	44 (38.6%)	46 (28.6%)	χ^2^ = 2.61, 0.106
Obesity II grade (n, %)	48 (42.1%)	51 (31.7%)	χ^2^ = 2.72, 0.099
Obesity III grade (n, %)	22 (19.3%)	64 (39.8%)	χ^2^ = 12.06, < **0.001**
WC (cm)	108.48 ± 15.16	119.69 ± 17.90	** < 0.001**
Blood pressure			
SBP (mmHg)	120.88 ± 9.41	136.80 ± 9.70	** < 0.001**
DBP (mmHg)	75.40 ± 7.32	86.54 ± 7.58	** < 0.001**
Metabolic parameters			
Fasting plasma glucose (mg/dL)	91.45 ± 10.41	108.30 ± 14.88	** < 0.001**
Fasting plasma insulin (μU/mL)	13.07 ± 16.17	18.33 ± 13.05	**0.003**
HoMA-IR	3.02 ± 3.80	5.10 ± 4.10	** < 0.001**
< 2.5 (n, %)	77 (67.5%)	46 (28.6%)	χ^2^ = 39.44, < **0.001**
> 2.5 (n, %)	37 (32.5%)	115 (71.4%)	
Total cholesterol (mg/dL)	195.61 ± 40.33	213.68 ± 38.02	** < 0.001**
LDL cholesterol (mg/dL)	121.93 ± 40.16	140.90 ± 37.76	** < 0.001**
HDL cholesterol (mg/dL)	50.37 ± 11.47	36.59 ± 7.62	** < 0.001**
Triglycerides (mg/dL)	116.57 ± 28.81	180.97 ± 42.65	** < 0.001**
Inflammatory parameters			
hs-CRP (mg/L)	1.37 ± 0.95	3.99 ± 2.69	** < 0.001**
Low cardiovascular risk (< 1 mg/L) (n, %)	49 (43.0%)	11 (6.8%)	χ^2^ = 49.04, < **0.001**
Intermediate cardiovascular risk (1–3 mg/L) (n, %)	59 (51.8%)	64 (39.8%)	χ^2^ = 43.42, 0.064
High cardiovascular risk (> 3 mg/L) (n, %)	6 (5.3%)	86 (53.4%)	χ^2^ = 67.37,< **0.001**
Nutritional parameters			
PREDIMED score	7.36 ± 1.65	4.62 ± 1.94	** < 0.001**
PREDIMED categories			
Low adherence to MD (n, %)	12 (10.5%)	118 (73.3%)	χ^2^ = 102.98, < **0.001**
Average adherence to MD (n, %)	93 (81.6%)	43 (26.7%)	χ^2^ = 78.21, < **0.001**
High adherence to MD (n, %)	9 (7.9%)	0 (0%)	χ^2^ = 10.76, **0.001**

Statistically significant values are shown in bold

Data are expressed as number and percentage or mean ± SD.

*BMI* body mass index, *WC* waist circumference, *SBP* systolic blood pressure, *DBP* diastolic blood pressure, *HoMA-IR* homeostasis model assessment insulin resistance *LDL* low-density lipoprotein, *HDL* high-density lipoprotein, *SD* standard deviation, *Hs-CRP* high sensitivity c reactive protein, *PREDIMED* PREvención con DIetaMEDiterránea, *MD* Mediterranean diet

MHO phenotype consumed more EVOO (p < 0.001), vegetables (p < 0.001), fruits (p < 0.001), wine (p = 0.008), legumes (p = 0.001), fish/seafood (p < 0.001), and tree nuts (p = 0.001) than MUO phenotype (Table 5). In addition, MHO phenotype reported a lower intake of red/processed meats (p < 0.001), butter, cream and margarine (p = 0.008), soda drinks (p = 0.006) and commercial sweets and confectionery (p = 0.002) compared to MUO phenotype.

**Table 5:** Response frequency of dietary components included in the PREDIMED questionnaire in subjects with MHO or MUO phenotype

Questions of PREDIMED questionnaire	MHO phenotype n = 114, 41.5%		MUO phenotype n = 161, 58.5%			
	n	%	n	%	χ^2^	*p-value
Use of EVOO as main culinary lipid	102	89.5	85	52.8	39.59	** < 0.001**
EVOO > 4 tablespoons	58	50.9	68	42.2	1.67	0.196
Vegetables ≥ 2 servings/day	79	69.3	56	34.8	30.45	** < 0.001**
Fruits ≥ 3 servings/day	61	53.5	40	24.8	21.01	** < 0.001**
Red/processed meats < 1/day	55	48.2	38	23.6	17.03	** < 0.001**
Butter, cream, margarine < 1/day	61	53.5	59	36.6	7.05	**0.008**
Soda drinks < 1/day	64	56.1	62	38.5	7.66	**0.006**
Wine glasses ≥ 7/week	61	53.5	59	36.6	7.05	**0.008**
Legumes ≥ 3/week	57	50.0	45	28.0	12.98	**0.001**
Fish/seafood ≥ 3/week	59	51.8	40	24.8	19.83	** < 0.001**
Commercial sweets and confectionery ≤ 2/week	47	41.2	37	23.0	9.63	**0.002**
Tree nuts ≥ 3/week	53	46.5	42	26.1	11.40	**0.001**
Poultry more than red meats	32	28.1	44	27.3	0.01	0.998
Use of sofrito sauce ≥ 2/week	50	43.9	69	42.9	0.01	0.966

Statistically significant values are shown in bold

Results are expressed as numbers and percentage.

*PREDIMED* PREvención con DIetaMEDiterránea, *MD* Mediterranean diet

*A p-value in bold type denotes a significant difference (p < 0.05).

In Table 6, the results of the bivariate proportional OR model performed to assess the association of MHO and MUO phenotype with dietary components of the PREDIMED questionnaire, PREDIMED score, and PREDIMED categories were summarized. The highest odds and R^2^ (indicative of high food consumption) of EVOO, vegetables and fruits, appeared to have a protective effect against MUO phenotype. A positive association was found between low adherence to MD and presence of MUO phenotype.

**Table 6:** Bivariate OR model to assess the association of subjects with MHO or MUO with the dietary components included in PREDIMED questionnaire, PREDIMED score and PREDIMED categories

Questions of PREDIMED questionnaire	OR	*p-value	95% IC	R^2^
Use of EVOO as main culinary lipid	0.13	** < 0.001**	0.067–0.258	0.152
EVOO > 4 tablespoons	0.71	0.157	0.436–1.144	0.007
Vegetables ≥ 2 servings/day	0.24	** < 0.001**	0.141–0.395	0.111
Fruits ≥ 3 servings/day	0.29	** < 0.001**	0.172–0.480	0.082
Red/processed meats < 1/day	0.33	** < 0.001**	0.198–0.556	0.064
Butter, cream, margarine < 1/day	0.50	**0.006**	0.308– 0.819	0.028
Soda drinks < 1/day	0.49	**0.004**	0.301–0.797	0.030
Wine glasses ≥ 7/week	0.50	**0.006**	0.308–0.819	0.028
Legumes ≥ 3/week	0.39	** < 0.001**	0.235–0.642	0.049
Fish/seafood ≥ 3/week	0.31	** < 0.001**	0.185–0.515	0.073
Commercial sweets and confectionery ≤ 2/week	0.43	**0.001**	0.252–0.718	0.037
Tree nuts ≥ 3/week	0.41	**0.001**	0.244–0.676	0.044
Poultry more than red meats	0.96	0.892	0.564–1.647	0.001
Use of sofrito sauce ≥ 2/week	0.96	0.869	0.592–1.558	0.001
PREDIMED score	0.46	** < 0.001**	0.377–0.548	0.346
PREDIMED categories				
Low adherence to MD	22.60	** < 0.001**	11.322–45.122	0.341
Average adherence to MD	0.08	** < 0.001**	0.046–0.148	0.267
High adherence to MD	0.01	0.999	0.000–0.001	0.057

OR is presented as a size effect for MUO. For PREDIMED categories, low, average and high adherence to MD were used as reference categories for comparison against the other variables.

*MUO* metabolically unhealthy obesity, *OR* odds ratio, *PREDIMED* PREvención con DIetaMEDiterránea, *MD* mediterranean diet

*A p-value in bold type denotes a significant difference (p < 0.05)

The PREDIMED score correlated positively with age (r = 0.143, p = 0.017) and HDL cholesterol (r = 0.441, p < 0.001) and negatively with BMI (r = − 0.369, p < 0.001), WC (r = − 0.392, p < 0.001), SBP (r = − 0.514, p < 0.001), DBP (r = − 0.506, p < 0.001), fasting plasma glucose (r = − 0.621, p < 0.001), fasting plasma insulin (r = − 0.359, p < 0.001), HoMA-IR (r = − 0.436, p < 0.001), total cholesterol (r = − 0.253, p < 0.001), LDL cholesterol (r = − 0.258, p < 0.001), HDL cholesterol (r = 0.441, p < 0.001), triglycerides (r = − 0,501, p < 0.001), and number parameters of MetS (r = − 0.703, p < 0.001) (Table 7).

**Table 7:** Correlations of PREDIMED score with age, anthropometric measurements, blood pressure, metabolic, inflammatory, and number of parameters of MetS

PREDIMED score (n = 275)		
Parameters	*r*	*p-value
Age (years)	0.143	**0.017**
Anthropometric measurements		
BMI (kg/m^2^)	− 0.369	** < 0.001**
WC (cm)	− 0.392	** < 0.001**
Blood pressure		
SBP (mmHg)	− 0.514	** < 0.001**
DBP (mmHg)	− 0.506	** < 0.001**
Metabolic parameters		
Fasting plasma glucose (mg/dl)	− 0.621	** < 0.001**
Fasting plasma insulin (μU/mL)	− 0.359	** < 0.001**
HoMA-IR	− 0.436	** < 0.001**
Total cholesterol (mg/dL)	− 0.253	** < 0.001**
LDL cholesterol (mg/dL)	− 0.258	** < 0.001**
HDL cholesterol (mg/dL)	0.441	** < 0.001**
Triglycerides (mg/dL)	− 0.501	** < 0.001**
Inflammatory parameter		
hs-CRP (mg/L)	− 0.681	** < 0.001**
Metabolic syndrome		
Number parameters (n)	− 0.703	** < 0.001**

*BMI* body mass index, *WC* waist circumference, *SBP* systolic blood pressure, *DBP* diastolic blood pressure, *HoMA-IR* homeostasis model assessment insulin resistance *LDL* low-density lipoprotein, *HDL* high-density lipoprotein, *SD* standard deviation, *Hs-CRP* high sensitivity c reactive protein

^*^A p-value in bold type denotes a significant difference (p < 0.05).

**Fig. 3:**
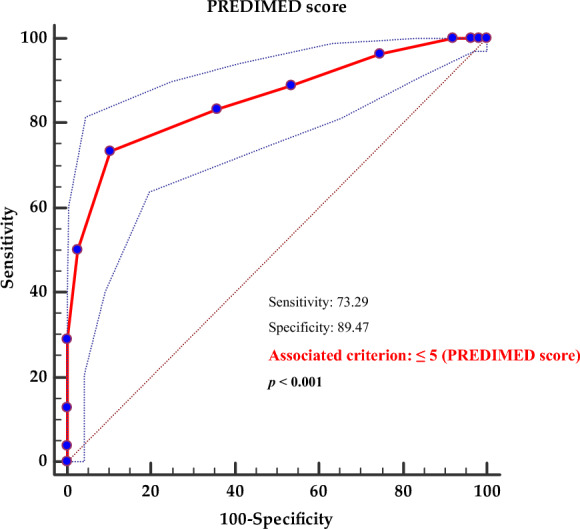
Receiver operator characteristic (ROC) analysis for PREDIMED score MUO phenotype predictive values. The 45-degree diagonal line functions as a benchmark, representing the ROC curve for random classification. The line marked with red dots illustrates the data distribution, while the two dashed lines surrounding it indicate the confidence intervals. The data point situated at the farthest northwest position on the ROC curve signifies the optimal threshold for classification. This threshold maximizes the accurate classification of subjects and minimizes instances of incorrect diagnoses. A bold p-value in bold indicates a significant difference (p < 0.05). PREDIMED, PREvención con DIetaMEDiterránea

According to ROC analysis, a PREDIMED score below 5 (p < 0.001, AUC 0.855, standard error 0.022, 95% CI 0.812 to 0.899) was identified as a cut-off point indicating a notably elevated likelihood of detecting the MUO phenotype; see Fig. 3.

At the logistic regression analysis between dietary components included in PREDIMED questionnaire and PREDIMED score, the latter (p < 0.001) and EVOO (p = 0.015) are those that contribute most to MHO/MUO phenotype (Table 8).

**Table 8:** Multiple regression analysis model (stepwise method) with the MHO/MUO phenotype as dependent variable to estimate the predictive value of the dietary components included in PREDIMED questionnaire and PREDIMED score

Parameters	Multiple regression analysis			
	*R*^*2*^	*β*	*t*	*p-value
PREDIMED score	0.352	-0.596	− 12.25	** < 0.001**
EVOO	0.364	-0.134	− 2.44	**0.015**

*PREDIMED* PREvención con DIetaMEDiterránea, *MD* mediterranean diet, *EVOO* extra virgin olive oil

^*^A p-value in bold type denotes a significant difference (p < 0.05)

## Discussion

In this cross-sectional observational study, we identified distinct differences between MHO and MUO phenotypes regarding their anthropometric and cardio-metabolic characteristics. As expected, MHO phenotype showed more favorable profiles such as BMI, waist circumference, blood pressure, insulin resistance, inflammation, and lower lipid levels than MUO phenotype. Moreover, our study contributed novel insights by providing a more detailed understanding of the dietary patterns of MHO and MUO phenotypes. Specifically, we observed that subjects with MHO displayed heightened adherence to MD, showing increased consumption of items such as EVOO, vegetables, fruits, legumes, fish, wine, and nuts in comparison to those with subjects with MUO. Additionally, subjects with MHO reported reduced consumption of red/processed meats, butter, cream, margarine, soda drinks, and commercial sweets and confectionery compared to their MUO counterparts. Significantly, the factors exerting the most substantial influence on the presence of MUO/MHO phenotype were adherence to MD and EVOO consumption. Notably, a PREDIMED score ≤ 5 was identified as the most sensitive and specific threshold for predicting the presence of MUO phenotype (in subjects with a BMI ≥ 30.0 kg/m^2^). These findings highlight the nuanced relationship between dietary habits and metabolic health, shedding light on factors that may contribute to differing phenotypes.

MD is a known widespread health-promoting dietary pattern; indeed, high adherence to MD has been associated with a low chance of developing obesity [30], insulin resistance [31] and type 2 diabetes mellitus [32]. In this context, it is conceivable to hypothesize that MD could also play a role in the context of MUO phenotype. In agreement with the findings of the current study, we previously found that women with MUO phenotype and polycystic ovary syndrome (PCOS) reported lower adherence to MD than women with MHO phenotype and PCOS, despite the same total energy intake [29]. Also, Leone et al. carried out a study in 2115 women with obesity that were divided according to MHO and MUO phenotypes [33]. Interestingly, they found that in post-menopausal state but not in premenopausal, higher adherence to MD was associated with a lower risk of MUO phenotype [33]. Also in adolescence, this association has been confirmed [34]. Indeed, a cross-sectional study was carried out in 203 adolescents with overweight/obesity. A validated food frequency questionnaire was administered in order to investigate dietary intakes, and an inverse association between MD and odds of MUO phenotype was found among Iranian adolescents [34]. The same results were found in the HELENA study, which was carried out in 137 adolescents with obesity or overweight in order to investigate the association of MUO phenotype with MD [35]. The authors found that adolescents with low adherence to MD had a higher likelihood of having MUO phenotype regardless of sex, age, energy intake, center, and body fat percentage [35]. Adherence to MD but also to Mediterranean-DASH intervention for neurodegenerative delay has been reported to be associated with a lower risk of MUO phenotype in subjects with obesity, as retrospectively observed over a mean of 5.91 years of follow-up [36]. These interesting results were definitively confirmed by a randomized controlled trial called the CORDIOPREV study that randomized subjects with obesity and coronary artery disease to receive Mediterranean or low-fat diets, finding that MD was not inferior to a low-fat diet in reducing the risk of developing MUO phenotype over the years [37].

Interestingly, we found that all the clusters of foods included in MD with a prominent effect of EVOO were associated with the risk of developing MUO phenotype. Numerous beneficial effects of MD could explain its role in promoting metabolic health. Several bioactive compounds introduced by following MD, such as polyphenols, mono- and polyunsaturated fatty acids, micronutrients, and antioxidants, contribute to metabolic health by their beneficial effects on inflammation and insulin resistance [38, 39]. In addition, MD decreases liver fat content and, thereby, improves glucose and lipid metabolism, also through regulation of hepatokine release [40, 41]. However, in our study, one of the central features of MD that seems to protect against MUO phenotype is EVOO. The high content of polyphenols in this nutrient confers antioxidant properties involved in the improvement of insulin resistance through the marked reduction of oxidative stress [42]. In light of these findings, the search for therapeutic strategies becomes imperative, particularly those incorporating the use of antioxidant-rich components of MD. MD, renowned for its antioxidant-rich components, has potential as a therapeutic avenue. By harnessing the antioxidant formulations inherent in Mediterranean foods, there is the prospect of mitigating the oxidative stress pathways linked to a spectrum of diseases, such as obesity [43].

Additionally, our investigation revealed that MUO phenotype, characterized by a lack of high adherence to MD, tended to consume a diet that contained an elevated proportion of energy-dense foods with limited nutritional value. This dietary pattern might lead to heightened consumption of refined carbohydrates, sugars, saturated fats, salt, and additives while having a reduced intake of fiber and essential micronutrients. Consequently, this dietary approach could contribute to the escalation of visceral fat accumulation and the initiation of low-grade inflammation, both established precursors for the development of insulin resistance [44, 45].

However, the identification of MHO and MUO phenotypes requires blood tests that can be time-consuming and costly and are not always performable on a large scale. Thus, the novelty of our study was to identify a threshold of PREDIMED score that can be easily detected carrying out a questionnaire to screen MHO/MUO phenotypes.

We are aware that there are some limitations to the current study. First, the cross-sectional design of this study did not allow for a causal information relationship. Second, PREDIMED score, although easy to perform by the participants, has the limit of being calculated based on a questionnaire. Thus, to avoid any bias related to self-report, PREDIMED questionnaire was administered face-to-face by the same nutritionist. Moreover, it's worth noting that PREDIMED questionnaire has been recently validated in different Mediterranean countries, including Italy [46]. However, we acknowledge the potential limitations when applying it to different ethnic groups, as generalization may still pose challenges. We did not analyze other inflammatory markers. Nevertheless, it is widely reported that hs-CRP represents the most studied inflammatory biomarker in different pathologic processes [47]. Finally, the study population exhibits an imbalance in terms of sex, with a predominance of female participants. Therefore, the proposed cut-off point of PREDIMED score for identifying MUO phenotype should be validated by further clinical trials.

## Conclusion

**Fig. 4:**
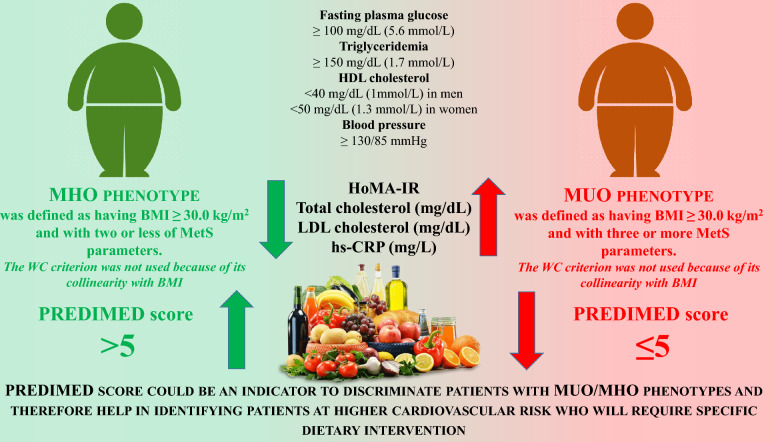
The summary results of this study. PREDIMED score could be an indicator to discriminate patients with MUO/MHO phenotypes and therefore help in identifying patients at higher cardiovascular risk who will require specific dietary intervention. HDL, High-Density Lipoprotein; MHO, Metabolic Healthy Obesity; MUO, Metabolic Unhealthy Obesity; BMI, Body Mass Index; MetS, Metabolic Syndrome; WC, Waist Circumference; PREDIMED, PREvención con DIetaMEDiterránea; HoMA-IR, Homeostasis Model Assessment Insulin Resistance LDL, Low-Density Lipoprotein; hs-CRP, High Sensitivity C Reactive Protein

In summary, our study highlights the significance of adhering to MD as a means to reduce the likelihood of developing MUO phenotype. The antioxidant properties of this nutritional pattern seem to be the main mechanism that explains this association. Thus, these data encourage the adoption of this nutritional pattern in obesity, mostly in subjects at high risk of MUO phenotype. In addition, the identification of a threshold PREDIMED score to screen MHO and MUO phenotypes could be easily used in an outpatient obesity clinic, thus allowing an immediate and inexpensive identification of these patients, potentially usable on a large scale. The summary results of the study are shown in Fig. 4.

## Data Availability

The datasets used and/or analyzed during the current study are available from the corresponding author on reasonable request.
